# Effect of Peptide Size on Antioxidant Properties of African Yam Bean Seed (*Sphenostylis stenocarpa*) Protein Hydrolysate Fractions

**DOI:** 10.3390/ijms12106685

**Published:** 2011-10-11

**Authors:** Comfort F. Ajibola, Joseph B. Fashakin, Tayo N. Fagbemi, Rotimi E. Aluko

**Affiliations:** 1 Department of Food Science and Technology, Federal University of Technology, Akure, Nigeria; E-Mail: comfort.funmilayo@yahoo.com (C.F.A.); 2 Department of Human Nutritional Sciences and the Richardson Centre for Functional Foods and Nutraceuticals, University of Manitoba, Winnipeg, MB, R3T 2N2, Canada

**Keywords:** African yam bean, enzymatic protein hydrolysate, ultrafiltration, antioxidant properties, peptide size, butylated hydroxyl toluene, glutathione

## Abstract

Enzymatic hydrolysate of African yam bean seed protein isolate was prepared by treatment with alcalase. The hydrolysate was further fractionated into peptide sizes of <1, 1–3, 3–5 and 5–10 kDa using membrane ultrafiltration. The protein hydrolysate (APH) and its membrane ultrafiltration fractions were assayed for *in vitro* antioxidant activities. The <1 kDa peptides exhibited significantly better (*p* < 0.05) ferric reducing power, diphenyl-1-picryhydradzyl (DPPH) and hydroxyl radical scavenging activities when compared to peptide fractions of higher molecular weights. The high activity of <1 kDa peptides in these antioxidant assay systems may be related to the high levels of total hydrophobic and aromatic amino acids. In comparison to glutathione (GSH), the APH and its membrane fractions had significantly higher (*p* < 0.05) ability to chelate metal ions. In contrast, GSH had significantly greater (*p* < 0.05) ferric reducing power and free radical scavenging activities than APH and its membrane fractions. The APH and its membrane fractions effectively inhibited lipid peroxidation, results that were concentration dependent. The activity of APH and its membrane fractions against linoleic acid oxidation was higher when compared to that of GSH but lower than that of butylated hydroxyl toluene (BHT). The results show potential use of APH and its membrane fractions as antioxidants in the management of oxidative stress-related metabolic disorders and in the prevention of lipid oxidation in food products.

## 1. Introduction

During respiration in aerobic organisms, reactive oxygen species or free radicals are generated as byproducts. Although these free radicals can exert diverse functions like signaling roles and providing defense against infections, excessive production can lead to oxidative stress. Oxidative stress has been associated with the development of chronic diseases such as cancer, atherosclerosis, coronary heart disease, diabetes, neurological malfunctioning and weakening of the immune system [1]. Although the mammalian body has physiological defense mechanisms to combat and reduce oxidative damage, these systems may not sufficiently protect the body against oxidative damage during severe oxidative stress. Hence, there is a need to supply the body with additional antioxidant. In addition, free radical mediated oxidation also negatively impact flavor, texture, nutritive value and shelf life of food products and under extreme conditions, produce toxins [2]. Thus, antioxidants are also important to the functional foods and nutraceutical industry.

The findings of numerous studies have confirmed that protein hydrolysates from enzymatic hydrolysis of animal and plant proteins can act as direct scavengers of diverse free radicals or behave as antioxidants in model systems [3–5]. In recent years, the antioxidant activities of enzymatic hydrolysates from plant-derived proteins, including soybean [6], wheat [5], chickpea [7], maize [1], canola [8], hemp seed [3,9], pea seed [4], flaxseed [10], buckwheat [11], peanut [12], alfalfa leaf [13], sesame seed [14] and rapeseed [15] have been evaluated using several *in vitro* antioxidant evaluation systems such as diphenyl-1-picryhydradzyl (DPPH), metal chelation, superoxide radical, hydroxyl radical, ferric reducing, and linoleic acid oxidation. The antioxidant properties of these hydrolysates largely depend on type of native protein and the operational condition applied to isolate the protein, specificity of the protease used for hydrolysis, degree of hydrolysis (DH), peptide structure, amino acid composition of the peptides and molecular weight of the peptides [11,16]. Hence, enzymatically modified proteins could be used as natural antioxidants to protect the human body against oxidative damage and associated disease. These protein hydrolysates may also serve as natural sources of antioxidants in functional foods to maintain freshness and extend shelf-life.

African Yam Bean (AYB) belongs to the family, *Papilionacea,* which is sometimes classified in the sub-family *Leguminosae*. It is one of the under-utilized legumes cultivated in various part of Africa for its edible seeds and potato-like spindle shaped tubers [17]. The economic potentials of AYB are immense because in addition to the production of two major food substances, the value of proteins in tubers and seeds is comparatively higher than what could be obtained from most African tuberous and leguminous crops [18]. The AYB tuberous roots have protein content varying from 11 to 19%. The seeds have protein content which ranged from 21.0 to 29.0% with about 50% carbohydrate mainly as starch [19]. The protein content of AYB seeds is however lower than soybean seed (38%), but amino acid spectrum indicated that lysine and methionine (limiting amino acid in most vegetable seed proteins) contents are better those of most legumes including soybean [20].

A review of available literatures on the previous work carried out on AYB seed revealed that there is scanty information on the antioxidant properties of AYB seed protein hydrolysates, suggesting the potential for development of value-added products from AYB seeds. Therefore preparation of antioxidant peptides from AYB seed proteins could be one way of producing high-value ingredients from this underutilized legume seed. The objectives of this work were to produce alcalase treated AYB seed protein hydrolysate, fractionate the hydrolysate into peptides of different molecular sizes and evaluate these samples for *in vitro* antioxidant properties using various antioxidant evaluation systems. Glutathione (GSH) was used for comparison purpose since it is a peptide and has physiological relevance as a cellular antioxidant molecule in human tissues.

## 2. Results and Discussion

### 2.1. Amino Acid Composition

The biological activity of a peptide is widely recognized to be based on the amino acid composition [21]. The amino acid compositions of AYB protein isolate (API), protein hydrolysate (APH) and membrane fractions are shown in Table 1. Glutamic acid + glutamine, aspartic acid + asparagine, *Gly*, *Leu*, *Lys* and *Ala* were the most predominant amino acids in API, APH and the membrane fractions. Hydrolysis of API with alcalase did not appreciably change the amino acid content of the hydrolysates and its membrane fractions. However, fractionation resulted in decreased level of *Cys* when compared to API and APH. *Ala*, *Met*, *Leu*, and *Trp* were highest in the <1 kDa fraction when compared to the other membrane fractions. In contrast, the <1 kDa peptides had less contents of Glutamic acid + glutamine, and aspartic acid + asparagine when compared to the other membrane fractions. The <1 kDa peptides also had the least content of *His*, *Lys*, and *Pro* when compared to the API, APH and other membrane fractions. Overall, the total hydrophobic amino acid (HAA) and aromatic amino acid (AAA) contents in 1 kDa peptide fraction were found to be higher when compared to those in API, APH and the other three fractions. For protein hydrolysates and peptides, an increase in hydrophobicity would increase their solubility in lipids and therefore, may enhance their antioxidative activity [5,22]. Some amino acids with aromatic and bulky side groups are strongly believed to contribute to the strong radical scavenging activities of peptides. For example, the ability of *His* (imidazole group) [23], *Trp* (indolic group) and *Tyr* (phenolic group) [24] to act as hydrogen donators have been attributed to the special groups they possess in their side chain. Aromatic amino acids (*Try* and *Phe*) have the ability to donate protons easily to electron deficient radicals while at the same time maintaining their stability via resonance structures. In addition, *Met* and *Cys* have the ability to donate their sulfur hydrogen; hence, these amino acids are considered effective radical scavengers [22].

### 2.2. DPPH Radical Scavenging Activities

**Figure 1:**
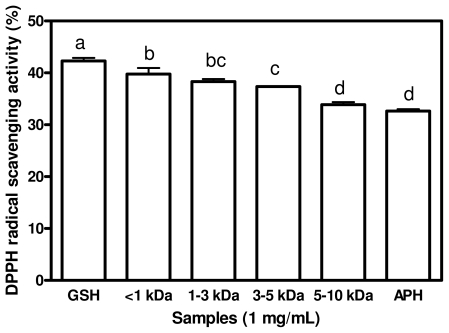
DPPH radical scavenging activities of African yam bean seed protein hydrolysate (APH) and its ultrafiltration membrane fractions.

DPPH radical is an oil-soluble free radical that becomes a stable product after accepting an electron or hydrogen from an antioxidant. DPPH radical is stable in methanol and show maximum absorbance at 517 nm. When DPPH encounters a proton-donating substance such as an antioxidant, the radical would be scavenged and the absorbance is reduced. Hence the antioxidant activity of the substance can be expressed as its ability in scavenging the DPPH radical. Figure 1 shows results of ability of APH and its membrane fractions to scavenge DPPH radical. GSH had the highest DPPH scavenging activity (42.29%) when compared to those of APH and its membrane fractions. There was no significant difference (*p* > 0.05) between the DPPH scavenging activities of <1 kDa and <3 kDa peptides. The result indicates that the DPPH radical Scavenging activities of the peptides are molecular size dependent. The lower -molecular weight (LMW) peptides, especially <1 kDa exhibited better DPPH radical scavenging activities than the high-molecular weight (HMW), especially the 3–5 kDa and 5–10 kDa peptides. This result is in agreement with the previous results reported by Girgih *et al.* [3] and also by Aluko and Monu [25], which showed that LMW peptide fractions had higher DPPH scavenging activities than HMW peptides. Increased hydrophobic character of peptides derived from protein sources have been shown to correlate with higher DPPH or other radical scavenging activities [4,7] when compared with peptide fractions of lower hydrophobic content. The highest DPPH radical scavenging activities that was found in the <1 kDa fraction may be due to its having the highest contents of total HAA and AAA. Kim *et al*. [26] reported that hydrophobic amino acids act as antioxidants by increasing the solubility of peptides in non-polar environments thereby facilitating better interaction with free radicals in order to terminate their activities. The results suggest that the high DPPH scavenging properties of LMW peptides could make them useful ingredients to that can be used to prevent oxidative deterioration of foods.

### 2.3. Superoxide Radical Scavenging Activities

**Figure 2:**
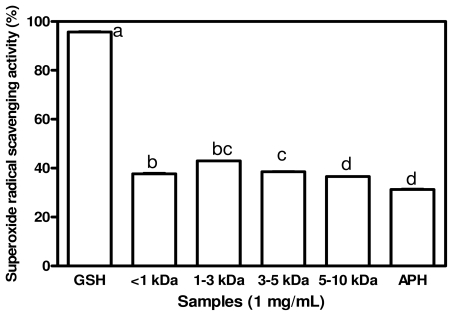
Superoxide radical scavenging activities of African yam bean seed protein hydrolysate (APH) and its ultrafiltration membrane fractions.

Superoxide radicals are generated by a number of biological reactions. Although superoxide radicals do not directly initiate lipid oxidation, they could promote oxidative reactions due to its ability to reduce transition metals, release protein-bond metals and form perhydroxyl radicals which initiate lipid oxidation [27]. For cytoprotection against this reactive oxygen, superoxide dismutase (SOD), which catalyzes the neutralization of superoxide anion to hydrogen peroxide, is one of the defense mechanisms in the living cell. Not only superoxide anion radicals but also their derivatives are celldamaging, which can cause damages to the DNA and cell membrane lipids [28]. Therefore, it is of great importance to scavenge superoxide anion radicals. The results of superoxide scavenging activities of APH and its membrane fractions are shown in Figure 2. GSH was significantly (*p* < 0.05) a more effective superoxide scavenger than the APH and peptide fractions. The results indicate that APH and its membrane fractions displayed moderate superoxide scavenging activity with the <3 kDa exhibited the significantly strongest (*p* < 0.05) superoxide activity of 43% and the APH exhibited the lowest superoxide scavenging activity of 31.3%. These scavenging activities are similar to those reported for chickpea protein hydrolysate (35–69%) [7] at 2.0 mg/mL peptide concentration while the present results were obtained at 1 mg/mL peptide concentration. In addition, Li *et al*. [7] also reported that a low molecular weight fraction from chickpea protein hydrolysate with strong superoxide radical scavenging activity was observed to have higher concentrations of *Phe*, *Ile*, *Leu* and *Val* in comparison to other fractions and it was therefore suggested that the superoxide scavenging activity was related to the hydrophobic amino acid. However, in the present work, there was no strong relationship between hydrophobic amino acid content and the superoxide scavenging activity of the peptide fractions. The results suggest that the peptides may not be as effective as GSH for the inhibition of superoxideinduced damage to cells. However, depending on degree of bioavailability, the peptides could offer some level of protection to cells against the toxic effects of superoxide free radicals.

### 2.4. Hydroxyl Radical Scavenging Activities

**Figure 3:**
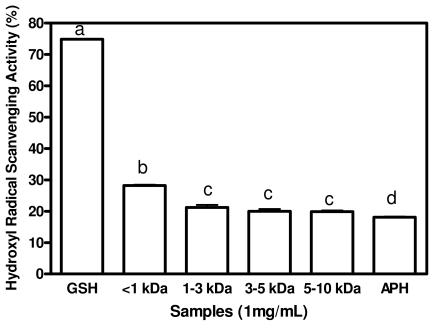
Hydroxyl radical scavenging activities of African yam bean seed protein hydrolysate (APH) and its ultrafiltration membrane fractions.

Among the oxygen radicals specifically, the hydroxyl radical is the most reactive specie and can be formed from superoxide anion and hydrogen peroxide, in the presence of metal ions, such as copper or iron. When a hydroxyl radical reacts with aromatic compounds, it can add onto a double bond, resulting in hydroxycyclohexadienyl radical. The resulting radical can undergo further reactions, such as reaction with oxygen, to give peroxyl radical, or decompose to phenoxyl-type radical by eliminating water [29]. Hydroxyl radical could severely damage adjacent biomolecules such as all proteins, DNA, polyunsaturated fatty acids, nucleic acids and almost all biological molecules that it comes in contact with. These hydroxyl radical-mediated damages can lead to premature aging, and the development of cancer and several diseases [5]. Therefore, removal of hydroxyl radical is one of the most effective defenses of a living body against various diseases. Hydroxyl radical scavenging activity of APH and its membrane fractions is shown in Figure 3. The result shows that <1 kDa displayed significantly strongest (*p* < 0.05) hydroxyl radical scavenging activity of 28.21% when compared to 1–3 kDa, 3–5 kDa, 5–10 kDa and APH. APH and its membrane fractions exhibited significantly lower (*p* < 0.05) hydroxyl radical activity when compared to that of GSH. Thus, GSH will likely provide better protection (when compared to peptides) to cells against damage by hydroxyl radicals. Since some degree of scavenging was obtained for the peptides, they have the potential to be developed as hydroxyl radical scavengers within cells. A number of studies have demonstrated a good correlation between certain amino acids residue or short peptides with radical scavenging activity of protein hydrolysates or peptides. Silver carp protein hydrolysates derived from alcalase was reported to possess stronger hydroxyl radical scavenging activity and contained higher concentrations of hydrophobic amino acids in comparison to a flavourzyme hydrolysate [30]. Li *et al*. [7] reported that a lower molecular weight fraction from chickpea protein hydrolysate with strong hydroxyl radical scavenging activity was observed to have higher concentration of hydrophobic amino acid, a result that is similar to the present result. Pownall *et al*. [4] also reported a strong correlation between the hydroxyl radical scavenging activity and total percentage of hydrophobic acid of the pea protein hydrolysate factions. Thus, the small peptide size in conjunction with high contents of HAA and AAA may have contributed to the superior hydroxyl radical scavenging property of the <1 kDa peptides when compared to the other peptide fractions with >1 kDa peptide size.

### 2.5. Metal Chelating Activity

**Figure 4:**
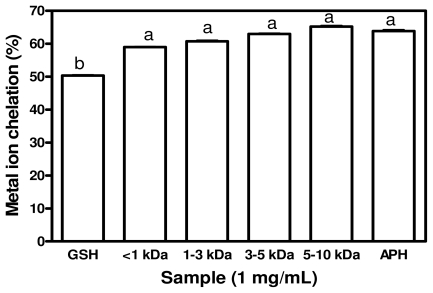
Metal chelating effects of African yam bean seed protein hydrolysate (APH) and its ultrafiltration membrane fractions.

Transition metal ions, such as Fe^2+^ and Cu^2+^ can catalyze the generation of reactive oxygen species which accelerates lipid oxidation. Fe^2+^ can also catalyze the Haber-Weiss reaction and induce superoxide anions to form more hazardous hydroxyl radicals. These hydroxyl radicals react with adjacent biomolecules to cause severe tissue damage [13], especially lipid oxidation. Therefore, the chelation of transition metal ions by antioxidative peptides could retard the oxidation reaction. Figure 4 shows the ability of APH and its membrane faction to chelate the transition metal ion, Fe^2+^. APH and its membrane fractions displayed similar metal chelating activities which were significantly stronger (*p* < 0.05) than that of GSH. The chelating activity of peptides in hydrolysates could enhance ability of tissues to reduce rate of deteriorative metal-catalyzed lipid oxidation. The peptides may also serve as good agents to prevent metal ion-dependent oxidative damage to food lipids and thereby serve as food preservatives. Carboxyl and amino group in the side chains of the acidic (*Glx* and *Asx*) and basic (*Lys*, *His* and *Arg*) amino acids are thought to play an important role in chelating metal ions [31]. Presumably, peptide cleavages lead to enhanced Fe^2+^ binding due to increased concentration of carboxylic (COO^−^) and amine groups in acidic and basic amino acids, thus removing the pro-oxidative free metal ions from the hydroxyl radical system. *His* residues have also been reported to contribute to the metal chelating effect of protein hydrolysates, which is largely related to its imidazole ring [23]. Although the percentage of *His* was very low, glutamic acid + glutamine, aspartic acid + asparagine, *Gly*, *Leu*, *Lys*, *Ala* and *Phe* are the most predominant amino acids in APH and the membrane fractions. These amino acids could have contributed to the metal ion chelating activity observed for the protein hydrolysates.

### 2.6. Ferric Reducing Power Activity (FRAP)

**Figure 5:**
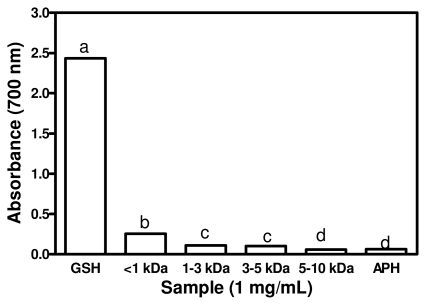
Ferric reducing power of African yam bean seed protein hydrolysate (APH) and its ultrafiltration membrane fractions.

The reducing capacity of a given compound may serve as a significant indicator of its potential antioxidant activity. An electron-donating reducing agent is able to donate an electron to a free radical. As a result, the radical is neutralized and the reduced species subsequently acquires a proton from the solution [32]. In this study, the reducing power of the APH and its membrane fractions was measured at the absorbance of 700 nm by transformation of the Fe^3+^/ferricyanide complex to ferrous form. An increased in absorbance indicate better reducing power of the test sample. Figure 5 shows that the reducing power activities of the APH and its membrane fractions were significantly lower (*p* < 0.05) than that of GSH. The 1 kDa fraction had significantly higher (*p* < 0.05) reducing power when compared to those of APH as well as the 1–3, 3–5, 5–10 kDa peptide fractions. The results indicate that smaller size peptides exhibited better reducing power than high molecular weight fractions. In contrast, Girgih *et al*. [3] reported an increase in reducing power of hemp seed protein hydrolysate with increasing peptide size. However, the present results are similar to the reducing power observed in smooth hound muscle protein hydrolysate and chickpea protein hydrolysate [7,33]. The correlations between the total hydrophobic amino acid and the reducing power of various hydrolysates have been reported [4]. Zhang *et al*. [15] reported that the rapeseed fractions that displayed the strongest reducing power also contained a higher amount of hydrophobic amino acids, which were suggested to be responsible for the strong reducing power of these peptides. The presence of amino acids such as *Leu*, *Lys*, *Met*, *Tyr*, *Ile*, *His* and *Trp* have been attributed to strong reducing power observed in protein hydrolysate fractions [34]. You *et al*. [35] suggested that the high amount of *Tyr*, *Met*, *His*, *Lys* and *Trp* that were found in the loach peptide hydrolysate could have contributed to the observed strong reducing power. However, recent report from multivariate analysis suggests that only sulphur-containing and acidic amino acids have strong contributions to the reducing power of food protein hydrolysates whereas positively charged and AAA may actually have negative effects [16]. The weak FRAP properties of the peptides indicate that they may not be as useful as GSH as electron donors when present within cells.

### 2.7. Inhibition of Linoleic Acid Oxidation

**Figure 6:**
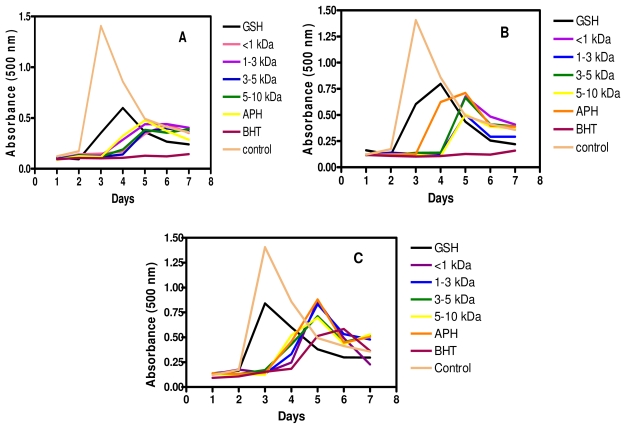
Inhibition of linoleic acid oxidation by African yam bean seed protein hydrolysate (APH) and its ultrafiltration membrane fractions. Concentrations of APH, peptide fractions, glutathione (GSH) and butylated hydroxyl toluene: **A**, 1 mg/mL; **B**, 0.5 mg/mL; **C**, 0.25 mg/mL.

Peroxidation of fatty acids can cause deleterious effects in foods and living tissues by forming complex mixtures of secondary break-down products of lipid peroxide. Further intake of these foods can cause a number of diverse effects including toxicity to mammalian cells [7]. Lipid peroxidation is thought to proceed via radical mediated abstraction of hydrogen atoms from methylene carbons in polyunsaturated fatty acids [22]. In ferric thiocyanate method, lipid oxidation products induce oxidation of ferrous iron to ferric iron which reacts with ammonium thiocyanate to from a color complex of ferric thiocyanate; therefore the absorption intensity is directly related to degree of linoleic acid oxidation [36]. As shown in Figure 6, the control (without hydrolysates) reached the highest absorbance on the third day, and gradually declined in the following 4 days. The rapid decline of the control after the third day was associated with the decomposition of (hydro) peroxide as the incubation time was increased [36]. The result agrees with the previous studies by Chen *et al*. [37], Li *et al*. [7] and Fasakin *et al*. [38]. The APH and its membrane fractions showed dose-dependent inhibition of lipid peroxidation in the linoleic acid emulsion system at 0.25–1.00 mg/mL peptide concentrations (Figures 6A–C). Generally as concentration of peptides increased from 0.25 mg/mL to 1.00 mg/mL, there were reductions in the maximum absorbance values, suggesting reductions in level of linoleic acid peroxidation. At 0.50 mg/mL peptide concentration, APH exhibited poorer inhibitory values in the first 4 days when compared to the fractionated peptides. However, at 1.00 mg/mL peptide concentrations there were no differences between the inhibitory activities of APH and fractionated peptides. Inhibitory activity of the APH and its membrane fractions was higher when compared to that of GSH but lower than that of butylated hydroxyl toluene (BHT), the two positive controls used in this experiment. Results reported in this study for GSH are similar to previous data, which showed that this compound gradually lost its ability to protect against linoleic acid oxidation after two days of incubation [3,4,38]. The decreased ability of GSH to inhibit lipid oxidation for a long period of time could be due to the fact that once GSH has been oxidized into GSSG, the regeneration of GSH (antioxidant form) is not possible as the experimental time increased. In contrast, the oxidation products of BHT also have antioxidant properties [39] and this could be responsible for the higher activity of BHT when compared to GSH, APH and its membrane fractions. Zhang *et al*. [40] reported that hydrophobic amino acids exhibited strong antioxidant activity to protect against lipid derivedradicals due to ability to interact with lipids. Hence the presence of hydrophobic amino acids in APH and its membrane fractions may have contributed to lipid peroxidation inhibitory activity by increasing lipid solubility of peptides and thereby facilitating better interaction with radical species. The high rate of inhibition of lipid oxidation provided by the peptides could be related to their high metal chelation properties, both of which are greater than the effects obtained with GSH. Therefore, the peptides could have roles as food preservatives by limiting lipid peroxidation and enhancing freshness. Depending on oral bioavailability, the peptides could also be used as inhibitors of lipid peroxidation, especially within blood vessels; such an action could help maintain regular blood flow by preventing deposition of lipid plaques. This could provide health benefits since lipid plaques within blood vessels can lead to restricted blood flow, hypertension and associated cardiovascular diseases.

## 3. Experimental Section

### 3.1. Materials

African yam bean seeds were purchased from Oba market, Omuo Ekiti, Ekiti State, Nigeria. Authentication of the seeds was carried out in the Department of Crop, Soil and Pest Management, Federal University of Technology, Akure, Nigeria. Alcalase, linoleic acid, DPPH, BHT, GSH, and other antioxidant reagents were purchased from Sigma (Sigma Chemicals, St. Louis, MO, USA) while other analytical grade reagents and ultrafiltration membranes (1, 3, 5, and 10 kDa molecular weight cut-offs) were obtained from Fisher Scientific (Oakville, ON, Canada).

### 3.2. Preparation of AYB Seed Protein Isolate (API)

API was produced from AYB seeds according to the method described by Adebowale *et al*. [41] with slight modifications. Briefly, AYB flour was dispersed in deionized water (1:20, w/v), and the dispersion was adjusted to pH 10.0 with 2 M NaOH to solubilize the proteins. The resultant dispersion was stirred at 37 °C for 2 h followed by centrifugation (7000 × g at 4 °C) for 1 h. The pellet was discarded, and the supernatant filtered with cheesecloth and adjusted to pH 5.0 with 2 M HCl to precipitate most of the proteins. Thereafter, the mixture was centrifuged (7000 × g at 4 °C) for 45 min, the resultant precipitate was re-dispersed in deionized water, adjusted to pH 7.0 with 2 M NaOH and freeze-dried to produce API powder. The protein content of API was determined using the modified Lowry method [42].

### 3.3. Preparation of AYB Protein Hydrolysate (APH) and Membrane Fractions

APH was produced according to the previous method reported by Omoni and Aluko [43]. Briefly, a 5% (w/v) API slurry was adjusted to pH 9.0 and heated to 50 °C followed by addition of alcalase (4% w/w, on the basis of protein content of API). The digestion was carried out for 4 h at 50 °C and reaction mixture was maintained at pH 9.0 by adding 2 M NaOH when necessary. The reaction was terminated by adjusting the mixture to pH 4.0 with 2 M HCl after which the reaction mixture was placed in boiling water for 15 min to ensure complete denaturation of enzyme protein and coagulation of undigested proteins. The mixture was allowed to cool to room temperature and centrifuged (7000 × g at 4 °C) for 30 min and the resulting supernatant (APH) was sequentially passed through ultrafiltration membranes with molecular weight cut-off (MWCO) of 1, 3, 5, and 10 kDa in an Amicon stirred ultrafiltration cell. Thus, the retentate from 1 kDa membrane was passed through 3 kDa membrane whose retentate was passed through 5 kDa and the last retentate was then passed through 10 kDa membrane to give permeates that have peptide sizes of <1, 1–3, 3–5, and 5–10 kDa, respectively. The permeate from each MWCO membrane was collected, lyophilized, and stored at −20 °C until needed for further analysis. The protein contents of the freeze-dried APH and membrane fractions were determined using the modified Lowry method [42].

### 3.4. Amino Acid Analysis

An HPLC system was used to determine the amino acid profiles after samples were hydrolyzed for 24 h with 6 M HCl according to the method previously described by Bidlingmeyer *et al*. [44]. The cysteine and methionine contents were determined after performic acid oxidation [45], and tryptophan content was determined after alkaline hydrolysis [46].

### 3.5. DPPH Radical Scavenging Assay

The scavenging activity of APH and its fractions against DPPH was determined as previously described [4] using a 96-well clear flat bottom plate. Peptide fractions were dissolved in 0.1 M sodium phosphate buffer, pH 7.0 containing 1% (w/v) Triton X-100. DPPH was dissolved in methanol to a final concentration of 100 μM. A blank control consisted of only DPPH and sodium phosphate buffer. Appropriate dilutions of the samples (100 μL) were mixed with 100 μL of DPPH solution in the 96-well plate to a final assay concentration of 1 mg/mL and incubated at room temperature in the dark for 30 min. Thereafter, the absorbance of the sample (As) and control (Ac) was read at 517 nm. The scavenging activity of the peptide fractions was compared to that of GSH (1 mg/mL). The percent scavenging activity of GSH and the samples was calculated using the following equation:

DPPH Radical Scavenging Activity (%)=(Ac-As/AC)×100

### 3.6. Assay of Metal Ion Chelation

The metal chelating activity was measured using a slightly modified version of a previous method Pownall *et al*. [4]. Peptide sample solution or GSH (final concentration of 1 mg/mL) was combined with 0.05 mL of FeCl_2_ (2 mM) and 1.85 mL distilled water in a reaction tube. Thereafter, 0.1 mL of 5 mM Ferrozine [3-(2-pyridyl)-5,6-diphenyl-1,2,4-triazine-4′,4″-disulfonic acid sodium salt] solution was added and mixed thoroughly. The mixture was allowed to stand at room temperature for 10 min followed by removal of 200 μL aliquot of the reaction mixture and added to a clear bottom 96-well plate. The control experiment contained all the reaction mixtures except that distilled water was used to replace the peptide. Absorbance of sample (As) and control (Ac) was measured using a spectrophotometer at 562 nm and the metal chelating activity of the sample was compared to that of GSH. The percentage chelating effect (%) was calculated using the following equation:

Metal chelating effect (%)=(Ac-As/Ac)×100

### 3.7. Ferric Reducing Power Assay

Reducing power was measured according to a method reported by Girgih *et al*. [3] with slight modifications. Peptide samples (250 μL) or GSH was prepared in 0.2 M sodium phosphate buffer (pH 6.6), mixed with 250 μL of buffer and 250 μL of 1% potassium ferricyanide solution dissolved in distilled water. The final peptide or GSH concentration in the assay mixture was 1 mg/mL while the control reaction contained buffer and ferricyanide only. Each resulting mixture was heated at 50 °C and incubated for 20 min, which was followed by addition of 250 μL 10% aqueous trichloroacetic acid. Subsequently, a 250 μL aliquot of the reaction mixture was combined with 50 μL of 0.1% aqueous ferric chloride solution and 200 μL of distilled water was added. The mixture was allowed to stand at room temperature for 10 min and then centrifuged at 1,000 × g for 10 min to obtain a supernatant, which was used to determine absorbance at 700 nm.

### 3.8. Hydroxyl Radical Scavenging Assay

The hydroxyl radical scavenging assay was modified based on a method described by Girgih *et al*. [3]. APH, peptide fractions, GSH and 1,10-phenanthroline (3 mM) were each separately dissolved in 0.1 M sodium phosphate buffer (pH 7.4) while FeSO_4_ (3 mM) and 0.01% hydrogen peroxide were each separately dissolved in distilled water. An aliquot (50 μL) of APH, peptide fractions or GSH (equivalent to a final assay concentration of 1 mg/mL) or buffer (control) was first added to a clear, flat bottom 96-well plate followed by additions of 50 μL of 1, 10-phenanthroline and 50 μL of FeSO_4_. To initiate reaction in the wells, 50 μL of hydrogen peroxide (H_2_O_2_) solution was added to the mixture, which was then covered and incubated at 37 °C for 1 h with shaking. Thereafter, the absorbance of the mixtures was measured at 536 nm every 10 min for a period of 1 h. The absorbance was also determined for a blank (does not contain peptides or H_2_O_2_) and a control (does not contain peptides). The OH^·^ scavenging activity was calculated as shown by described by Girgih *et al*. [3].

### 3.9. Superoxide Scavenging Assay

The superoxide radical scavenging activity was determined according to the method described by Pownall *et al*. [4]. An aliquot (80 μL) of peptide fraction or GSH (equivalent to a final assay concentration of 1 mg/mL) was mixed with 80 μL of 50 mM Tris–HCl buffer (pH 8.3) containing 1 mM EDTA in a clear bottom 96-well plate. Then, 40 μL of 1.5 mM pyrogallol (dissolved in 10 mM HCl) was added to each well. The reaction rate (Δ*A*/min) was monitored as increase in absorbance at 420 nm for 4 min at room temperature. The control mixture contained the buffer but no peptide or GSH was added. The superoxide radical scavenging activity of the samples was calculated as shown by Pownall *et al*. [4].

### 3.10. Inhibition of Linoleic Acid Oxidation

Linoleic acid oxidation was measured using a slight modification of the method described by Girgih *et al*. [3]. Peptide fractions or standard compounds (GSH and BHT) at final assay concentrations of 0.25, 0.5, and 1.0 mg/mL were each dissolved in 1.5 mL of 0.1 M sodium phosphate buffer, pH 7.0. Each mixture was added to 1 mL of 50 mM ethanolic linoleic acid and stored in a glass test tube kept at 60 °C in the dark for 7 days. On a daily basis, 100 μL of the sample mixture was removed and mixed with 4.7 mL of 75% aqueous ethanol, 0.1 mL of ammonium thiocyanate (30%, w/v) and 0.1 mL of 0.02 M acidified ferrous chloride (dissolved in 1 M HCl). An aliquot (200 μL) of the resulting solution was added to a clear bottom 96-well plate and the degree of color development was measured using the spectrophotometer at 500 nm after 3 min incubation at room temperature.

### 3.11. Statistics Analysis

Data were collected as means of 3 separate determinations and subjected to one way analysis of variance using Statistical Analysis System Software (SAS version 9.2, SAS institute, Cary, NC, USA). Significant differences between mean values were determined by Duncan’s multiple range tests and accepted at *p* < 0.05.

## 4. Conclusions

Results of this study showed that protein hydrolysates derived from African yam bean seed possess antioxidant properties against a variety of physiologically relevant free radicals. Small peptide size and high level of hydrophobicity seem to be important for scavenging of hydroxyl radical and DPPH radicals as well as Fe^3+^ reduction. The peptides had better metal chelating properties than GSH, which could have been responsible for the greater inhibition of lipid oxidation by peptides. This is highly significant because lipid oxidation is believed to promote development and growth of atherosclerotic plaques in blood vessels. Therefore, if these peptides are later shown to be orally bioavailable, they could provide effective means of preventing plaque formation within blood vessels of humans and may be used as agents against development of cardiovascular diseases. Overall, the effectiveness of APH and its membrane fraction to scavenge free radicals, chelate metals and inhibit linoleic acid oxidation suggests that these products possess potential as a food source of antioxidant agents. Hence, they could be used as raw materials for the production of peptide ingredients that can be used to formulate functional foods and nutraceuticals. They could also be used as natural source of antioxidants (preservatives) in the food industry to prevent lipid oxidation and maintain freshness during production and storage of food products.

## Figures and Tables

**Table 1:** Percentage amino acid compositions of African yam bean protein isolate, (API), protein hydrolysate (APH), and membrane ultrafiltration fractions.

	API	APH	<1 kDa	1–3 kDa	3–5 kDa	5–10 kDa
ASX	11.23	11.38	9.60	11.03	11.50	11.50
THR	3.92	4.06	4.54	4.07	3.63	3.79
SER	6.82	6.93	8.05	6.84	6.41	6.32
GLX	12.47	13.27	11.34	13.05	13.50	13.88
PRO	1.36	1.44	0.85	1.36	1.49	1.55
GLY	9.05	8.96	8.76	8.77	8.86	8.81
ALA	7.21	7.37	9.17	7.63	7.15	7.06
CYS	1.10	0.84	0.27	0.47	0.59	0.62
VAL	4.91	4.97	5.15	5.40	5.35	5.52
MET	1.20	0.96	1.13	0.92	0.97	0.97
ILE	4.14	3.96	3.73	4.29	4.33	4.41
LEU	8.56	8.35	10.25	9.11	8.70	8.47
TYR	4.54	3.73	3.85	3.65	3.66	3.59
PHE	6.03	5.97	7.34	6.34	6.02	5.96
HIS	3.48	3.59	2.99	3.33	3.59	3.64
LYS	8.03	8.33	7.36	8.01	8.26	8.10
ARG	4.90	4.97	4.45	4.90	5.14	5.03
TRP	1.04	0.94	1.17	0.84	0.85	0.80
HAA	40.09	38.52	42.91	40.01	39.11	38.94
PCAA	16.42	16.88	14.80	16.23	16.99	16.77
NCAA	23.70	24.65	20.94	24.08	25.00	25.38
AAA	11.62	10.64	12.36	10.82	10.53	10.35

ASX = aspartic acid + asparagine; GLX = glutamic acid + glutamine; Combined total of hydrophobic amino acids (HAA) = alanine, valine, isoleucine, leucine, tyrosine, phenylalanine, tryptophan, proline, methionine and cysteine; Positively charged amino acids (PCAA) = arginine, histidine, lysine; Negatively charged amino acids (NCAA) = ASX and GLX; Aromatic amino acids (AAA) = phenylalanine, tryptophan and tyrosine.
